# Anticancer effect of berberine based on experimental animal models of various cancers: a systematic review and meta-analysis

**DOI:** 10.1186/s12885-019-5791-1

**Published:** 2019-06-17

**Authors:** Jianhao Xu, Yuming Long, Liwei Ni, Xuya Yuan, Na Yu, Runhong Wu, Jialong Tao, Yusong Zhang

**Affiliations:** 1 Department of Pathology, Kunshan First People’s Hospital Affiliated to Jiangsu University, Kunshan, Jiangsu 215004 People’s Republic of China; 2 Department of Oncology, The Second Affiliated Hospital of Soochow University, Suzhou, Jiangsu 215004 People’s Republic of China

**Keywords:** Berberine, Cancer, Experimental animals, Meta-analysis

## Abstract

**Background:**

Numerous studies have explored the anti-tumor effect of berberine (BBR), but little clinical evidence guides the use of BBR in cancer patients.

**Objectives:**

Our aim was to investigate the impact of BBR on various cancers in healthy animals to promote the transformation from bench to bed.

**Search methods:**

PubMed, Embase, Springer, and Cochrane databases were searched from January 2000 to October 2018 for relevant articles.

**Selection criteria:**

Only published studies focusing on the relationship between BBR and various cancers in vivo were qualified. Two review authors independently assessed the risk of bias for each study, and any disagreement was resolved by discussion or by involving a third assessor.

**Results:**

A total of 26 studies from 2000 to 2018, focusing on various cancer types, including breast cancer, liver cancer, colorectal cancer, nasopharyngeal carcinoma, lung cancer, gastric cancer, neuroepithelial cancer, endometrial carcinoma, esophageal cancer, tongue cancer, cholangiocarcinoma, and sarcoma were included. Overall, BBR reduced tumor volume (SMD =3.72, 95% CI: 2.89, 4.56, Z = 8.73, *p* < 0.00001) and tumor weight (SMD =2.35, 95% CI: 1.51, 3.19, Z = 5.50, *p* < 0.00001) in a linear The dose–response relationship (Pearson r = − 0.6717, *p* < 0.0001 in tumor volume analysis; Pearson r = − 0.7704, *p* < 0.0005 in tumor weight analysis). BBR inhibited angiogenesis in tumor tissues (SMD = 4.29, 95% CI: 2.14, 6.44, Z = 3.92, *p* < 0.00001), but it had no significant effect on the body weight of experimental animals (SMD = 0.11, 95% CI: − 0.70, 0.92, Z = 0.27, *p* = 0.78). Publication bias was not detected.

**Conclusion:**

BBR exerted anti-tumor effects in a variety of tumors in vivo, especially breast cancer and lung cancer, and the evidence was still insufficient in colorectal cancer and gastric cancer.

## Background

Berberine (BBR) is a natural component purified from the species of the genus *Berberis*, which has long been used as an anti-diarrheal drug in gastrointestinal disorders in traditional Chinese medicine [1]. At the same time, the anti-tumor effect of BBR has been a hot topic in experimental research in recent years. In the past 3 years, latest studies have shown the anticancer actions of BBR against several high-risk cancers, including lung cancer [2], breast cancer [3], prostate cancer [4], colorectal cancer [5], and gastric cancer [6].

However, little clinical evidence guides the use of BBR in cancer patients. Thus, systematic reviews and meta-analyses of animal studies may help to clarify whether cancer patients could benefit from this approach and promote the transformation of animal studies into humans at the same time [7].

Our aim was to investigate the impact of BBR on cancer growth and its adverse effects in randomized controlled trials in healthy animals.

## Methods

### Identification of studies

From January 2000 to October 2017, relevant literature from PubMed, Embase, Springer, and Cochrane databases was systematically screened. The following Mesh terms and textwords were used: “Neoplasms”[Mesh], “Neoplasia,” “Neoplasias,” “Neoplasm,” “Tumors,” “Tumor,” “Cancer,” “Cancers,” “Malignant Neoplasms,” “Malignant Neoplasm,” “Neoplasm, Malignant,” “Neoplasms, Malignant,” “Malignancy,” “Malignancies,” “Berberine”[Mesh]," “Berberine,” “Umbellatine,” and “BBR.” The “AND” or “OR” operator was used to combine these terms in varying combinations. At the same time, references in the articles were also included in the screening. We did not set a language limit during the process. Two authors (Jianhao Xu, Yuming Long) independently reviewed the titles and abstracts identified in the search. In this process, we discussed the articles to incorporate the differences. If problems still could not be resolved, a third assessor (Yusong Zhang) was invited to make a decision. Only published articles were included. No protocol was developed for this review.

### Selection criteria

The inclusion criteria were as follows: (1) participants: experimental animals including rodent, mouse, rat, rabbit, guinea pig, dog, horse, sheep, and monkey; (2) invention: BBR only; (3) outcomes: the effects of BBR in animal models after tumor implantation, including tumor volume, tumor weight, tumor vessel density, and body weight; and (4) study design: experiments should be prospectively controlled. The exclusion criteria were as follows: (a) literature published as letters, editorials, abstracts, reviews, and expert opinions; (b) non-animal-based studies; (c) articles with missing data information; (d) similar and repeated studies; and (e) outdated articles with little significance and credibility. Cohen’s kappa statistic was used to assess chance-corrected agreement between reviewers (SPSS version 18. 0, SPSS Inc. Chicago, IL) [8].

### Study characteristics and data extraction

A detailed form was designed for data extraction: first author, publication year, country, cancer type, animals’ baseline characteristics, intervention, duration, and the data of specific outcomes (tumor volume, tumor weight, tumor vessel density, and body weight). Two review authors extracted the data by using the agreed form.

### Quality of evidence and risk of bias

For risk of bias of individual studies, the ARRIVE checklist was used to assess pre-clinical animal studies [9]. For risk of bias among studies, such as publication bias and selective reporting, funnel chart analysis, subgroup analysis, and sensitive analysis were all conducted. Two review authors (Jian−hao Xu, Yuming Long) independently assessed the risk of bias for each study.

### Data synthesis and statistical analysis

We carried out statistical analysis by using the Review Manager software (RevMan 5.3) and STATA statistical software package version 12.0 (Stata Corporation, College Station, TX). The primary outcomes were tumor volume, tumor weight, and tumor vessel density of BBR group compared with the control group. The secondary outcome was the change of body weight. Mean value and standard difference (SD) were used as summary statistics. Standard mean difference (SMD) was measured for continuous data. Linear regression and Pearson’s correlation analysis were used to study the The dose–response relationship between BBR and the four outcomes. The heterogeneity among studies was measured by using the *I*^2^ test. The latent publication bias was assessed by using a funnel plot. All statistical tests were two-tailed, and *p* < 0.05 was considered statistically significant.

## Results

### Search results

**Fig. 1:**
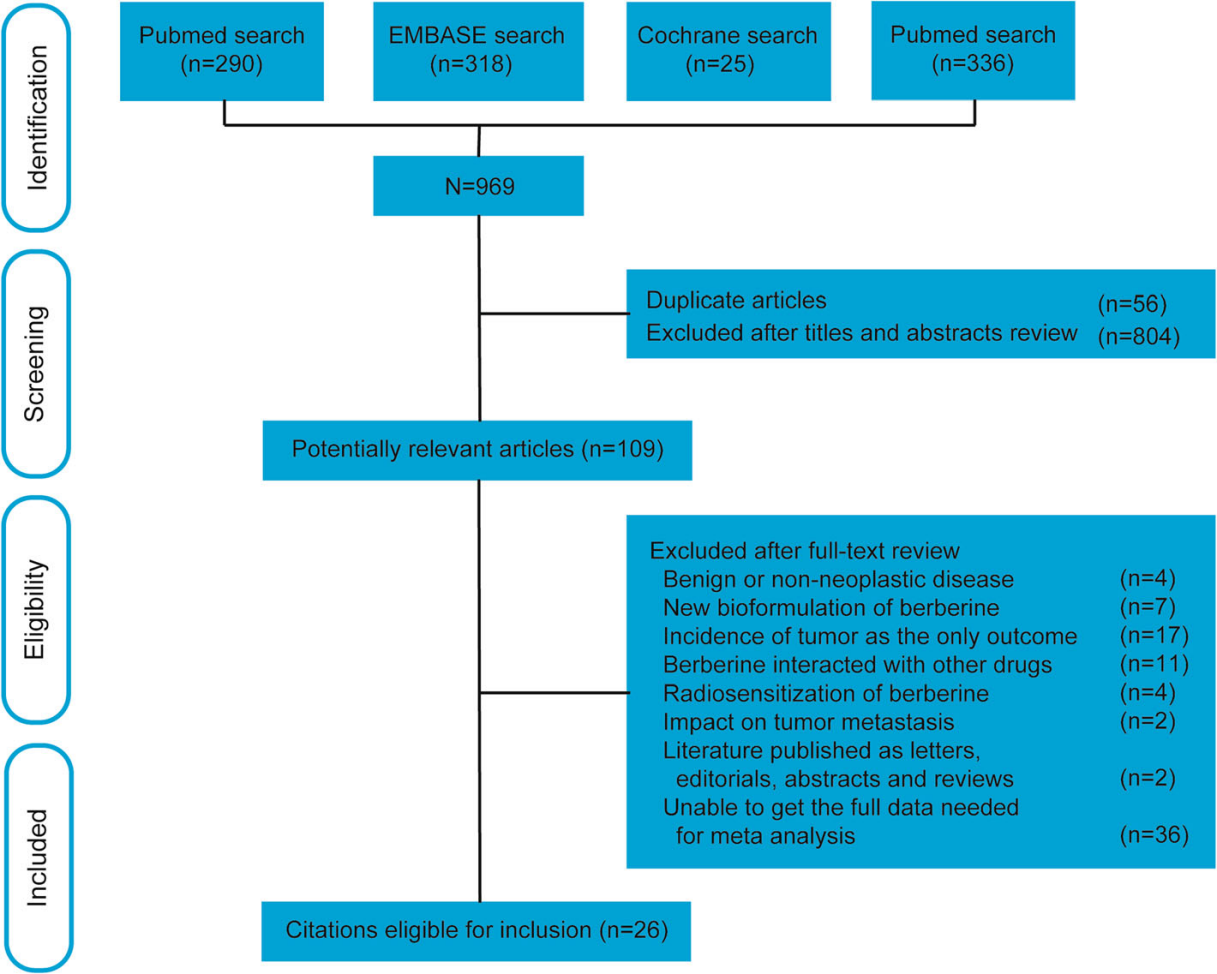
Flow chart for the selection of records to include

A total of 969 potential articles were identified from the literature search. After selection, 26 studies [10–35] matched the inclusion criteria and were suitable for our meta-analysis. The flow diagram in Fig. 1 showed the selection process. A review of the study selection and data extraction indicated excellent agreement between reviewers (k = 0.820).

### Study characteristics and quality assessment

Study characteristics are summarized in Table 1. A total of 26 studies [10–35] from 2000 to 2018, focusing on various cancer types, including breast cancer [10–16], liver cancer [17–19], colorectal cancer [20–22], nasopharyngeal carcinoma [23, 24], lung cancer [25, 26], gastric cancer [27, 28], neuroepithelial tumor [29, 30], endometrial carcinoma [31], esophageal cancer [32], tongue cancer [33], cholangiocarcinoma [34], and sarcoma [35] were included. The studies used rats [14], hamsters [34], and mice [10–13, 15–33, 35] modeled via subcutaneous tumor implantation [10–13, 16–35] or induced tumor formation [14, 15]. BBR was administered in doses ranging between 2.5 mg/kg and 200 mg/kg body weight through intraperitoneal injection [10, 15–17, 19, 21, 23, 24, 30, 33, 35] and gavage [11–14, 18, 20, 22, 25–29, 31, 32, 34] or from 1000 ppm to 5400 ppm in drinking water [13, 20, 25]. The size of the study sample ranged from 6 to 20, while the follow-up ranged from 1 week to 32.5 weeks. Quality assessment based on the ARRIVE guideline is presented in Table 2. Overall, the studies included in our analysis were of moderate quality.

**Table 1:** Characteristics of prospective studies on BBR

Author, year, country	Species, strain, gender, age	Model cell line	Experiment				Control	Outcome							
			Dosage	Frequency	Adnimistration	Duration		type	mean0	Sd0	n0	mean1	Sd1	n1	*p* value
Breast cancer															
Elisa Pierpaoli, 2015, Italy [10]	mice, FVB/N, F, 4w	SK-BR-3	2.5 mg/kg	biw	ip	32.5w	DMSO	VD	16.81	7.24	10	12.09	1.98	10	0.07
Yuwan Zhao, 2017, China [11]	mice, BALB/c, F, 6w	MDA-MB-231	100 mg/kg	tiw	po	3w	DMSO	TV	2.70	0.18	7	0.68	0.08	7	< 0.01
								BW	24.40	0.69	7	22.48	0.81	7	< 0.01
Alaa Refaat, 2013, Japan [12]	mice, BALB/c, F, 6w	4 T1	100 mg/kg	qd	po	4.3w	CMC	TW	0.21	0.01	6	0.15	0.01	6	< 0.01
Sangmin Kim, 2018, Korea [13]	mice, Balb/c, F, 6-8w	MDA-MB-231	0.1% BBR in the drinking water	Daily free intake	po	6.6w	–	TV	0.42	0.18	5	0.21	0.08	5	0.05
Kalyani Chowdary Karnam, 2017, India [14]	rats, SD, F, 6.4–8.3w	Induced by DMBA	50 mg/kg [pretreatment]	tiw	po	4w	Corn oil	TV	3.79	0.90	6	0.63	0.30	6	< 0.01
								TW	9.64	0.90	6	3.80	0.99	6	< 0.01
			50 mg/kg [posttreatment]					TV	3.79	0.90	6	1.31	0.60	6	< 0.01
								TW	9.64	0.90	6	5.71	1.32	6	< 0.01
Elisa Damiani, 2015, Italy [15]	miceFVB/NF4w	HER2/neu transgenic mice	2.5 mg/kg	biw	ip	NR	Sterile saline	VD	16.77	5.31	7	11.07	1.75	9	0.03
Ke Su, 2016, China [16]	mice, Balb/c, F, 6w	MDA-MB-231	10 mg/kg	q4d	ip	3w	DMSO	TV	0.59	0.27	6	0.27	0.12	6	0.02
								TW	0.50	0.11	6	0.29	0.06	6	< 0.01
								BW	22.59	7.31	6	19.10	3.71	6	0.32
Liver cancer															
Guan-Yu Wang, 2009, China [17]	mice, Balb/c, M, 6w	HEPG2	40 mg/kg	qd	ip	1.4w	Saline	TV	3.31	0.38	5	2.21	0.22	6	< 0.01
								BW	3.13	0.43	5	4.62	0.41	6	< 0.01
			80 mg/kg					TV	3.31	0.38	5	1.43	0.13	5	< 0.01
								BW	3.13	0.43	5	3.74	0.36	5	0.04
Jing Li, 2015, Canada [18]	mice, Balb/c, NR, 6-8w	H22	50 mg/kg	qd	po	2w	Water	TV	4.24	0.56	10	0.33	0.35	10	< 0.01
Chi Man Tsang, 2015, China [19]	mice, NR, NR, NR	MHCC-97 L-luciferase	10 mg/kg	qod	ip	5w	Saline	TV	1.00	0.05	7	0.21	0.03	7	< 0.01
								VD	12.58	2.94	7	2.18	1.29	7	< 0.01
Colon cancer															
Norio Iizuka, 2002, Japan [20]	mice, Balb/c, M, 6w	Colon26/clone 20	0.1% BBR in the driNRing water	Daily free intake	po	2w	–	TW	0.22	0.15	9	0.25	0.12	9	0.65
								BW	18.20	1.50	9	18.40	1.80	9	0.80
			0.2% BBR in the driNRing water					TW	0.22	0.15	9	0.25	0.15	9	0.68
								BW	18.20	1.50	9	22.20	1.50	9	< 0.01
			0.4% BBR in the driNRing water					TW	0.22	0.15	9	0.24	0.18	9	0.80
								BW	18.20	1.50	9	20.90	4.20	9	0.10
H Ruan, 2017, China [21]	mice, Balb/c, NR, 6-7w	KM12C/shCtrl	10 mg/kg	qd	ip	2w	DMSO	TV	1.26	0.97	6	0.79	0.53	6	0.32
		KM12C/shRXRα							1.54	0.92	6	1.40	0.46	6	0.76
Yuchen Cai, 2013, Japan [22]	mice, Balb/c, NR, 5w	HT-29	10 mg/kg	qd	po	2w	Sterile water	TV	6.11	3.01	10	4.33	2.42	10	0.16
								BW	6.60	3.60	10	4.90	3.20	10	0.28
			30 mg/kg					TV	6.11	3.01	10	4.09	1.76	10	0.08
								BW	6.60	3.60	10	3.90	2.70	10	0.07
			50 mg/kg					TV	6.11	3.01	10	3.34	1.31	11	0.01
								BW	6.60	3.60	10	3.60	2.50	11	0.04
nasopharyngeal carcinoma															
Chao Wang, 2017, China [23]	mice, NOD/SCID, F, 8w	HONE-1	10 mg/kg	tiw	ip	3w	DMSO	TV	0.58	0.06	5	0.10	0.03	5	< 0.01
								TW	0.15	0.01	5	0.02	0.01	5	< 0.01
Chi Man Tsang, 2013, China [24]	mice, NR, M, 6-8w	C666–1	5 mg/kg	qod	ip	6w	DMSO	TV	0.15	0.05	5	0.04	0.03	5	< 0.01
			10 mg/kg						0.15	0.05	5	0.02	0.02	4	< 0.01
Lung cancer															
Michael A. James, 2011, Missouri [25]	mice, Balb/c, M, 4-6w	A549	1800 ppm	Daily free intake	po	4w	DMSO	TV	0.06	0.02	3	0.02	0.02	4	0.05
			5400 ppm						0.06	0.02	3	0.01	0.01	2	0.04
Santosh K. Katiyar, 2009, Alabama [26]	mice, Balb/c, F, 6-7w	A549	50 mg/kg	qd	po	7w	PBS	TV	1.40	0.07	10	0.99	0.04	10	< 0.01
								TW	2.32	0.27	10	2.02	0.30	10	0.03
			100 mg/kg					TV	1.40	0.07	10	0.60	0.03	10	< 0.01
								TW	2.32	0.27	10	1.16	0.21	10	< 0.01
			200 mg/kg					TV	1.40	0.07	10	0.30	0.06	10	< 0.01
								TW	2.32	0.27	10	0.62	0.09	10	< 0.01
		H1299	50 mg/kg					TV	1.59	0.10	10	1.36	0.05	10	< 0.01
								TW	2.71	0.31	10	2.36	0.29	10	0.02
			100 mg/kg					TV	1.59	0.10	10	1.05	0.05	10	< 0.01
								TW	2.71	0.31	10	1.82	0.29	10	< 0.01
			200 mg/kg					TV	1.59	0.10	10	0.61	0.02	10	< 0.01
								TW	2.71	0.31	10	1.15	0.10	10	< 0.01
Gastric cancer															
Junxiong Wang, 2016, China [27]	mice, Balb/c, F, 5w	BGC823	50 mg/kg	qd	po	4w	NR	TV	2.28	0.24	3	0.73	0.13	3	< 0.01
								TW	1.37	0.37	3	0.32	0.08	3	< 0.01
								BW	2.51	0.69	3	0.10	0.46	3	< 0.01
Hongli Li, 2016, China [28]	mice, Balb/c, M, 4w	MGC803	15 mg/kg	qd	po	3.3w	NR	TV	0.85	0.29	6	0.44	0.09	6	0.02
								TW	0.68	0.18	6	0.42	0.07	6	< 0.01
Neuroepithelial tumor															
Juan Wang, 2015, China [29]	miceBalb/cNN	–	100 mg/kg	qd	po	3w	NR	TV	0.04	0.02	8	0.02	0.00	8	0.05
Yuxue Sun, 2018, China [30]	miceBalb/cN6-8w	C6	10 mg/kg	qd	ip	1w	DMSO	TV	0.77	0.22	7	0.35	0.06	7	< 0.01
Endometrial carcinoma															
Yu Wang, 2018, China [31]	mice, Balb/c, NR, 6w	HEC-1-A	50 mg/kg	qd	po	4w	DMSO	TV	1.01	0.13	6	0.65	0.06	6	< 0.01
			100 mg/kg						1.01	0.13	6	0.34	0.04	6	< 0.01
Esophageal cancer															
Kewei Ren, 2016, China [32]	mice, Balb/c, M, 6-8w	Eca9706	20 mg/kg	qd	po	7w	DMSO	TV	6.37	0.25	5	5.05	0.60	5	< 0.01
								TW	2.66	0.29	5	1.82	0.21	5	< 0.01
Tongue squamous cell carcinima															
Yung-Tsuan Ho, 2009, China [33]	mice, Balb/c, F, 6w	SCC-4	10 mg/kg	q4d	ip	4w	DMSO	TV	0.18	0.06	6	0.03	0.02	6	< 0.01
								TW	0.26	0.16	6	0.12	0.09	6	0.11
Cholangiocarcinoma															
Nattapong Puthdee, 2013, Japan [34]	hamster, Syrian, M, 4-5w	Ham-1	10 mg/kg	qd	po	3w	sterile water	TW	0.70	0.18	5	0.67	0.11	5	0.79
Sarcoma															
Lei Zhang, 2012, China [35]	mice, Kunming, NR, 6w	S180	30 mg/kg	qd	ip	NR	NR	TW	2.20	0.93	10	1.26	0.54	9	0.02
								BW	2.71	2.20	10	−2.54	3.24	9	< 0.01

Mean0: mean value in control group (cm^3^ for tumor volume, g for tumor weight and body weight, mm/mm^2^ for vessel density); Sd0: standard difference in control group; N0: sample size in control group; Mean1: mean in berberine group; Sd1: standard difference in berberine group; N1: sample size in berberine group; M: male; F: femle; NR: non reported; TV: tumor volume; TW: tumor weight; VD: vessel density; BW: body weight

**Table 2:**
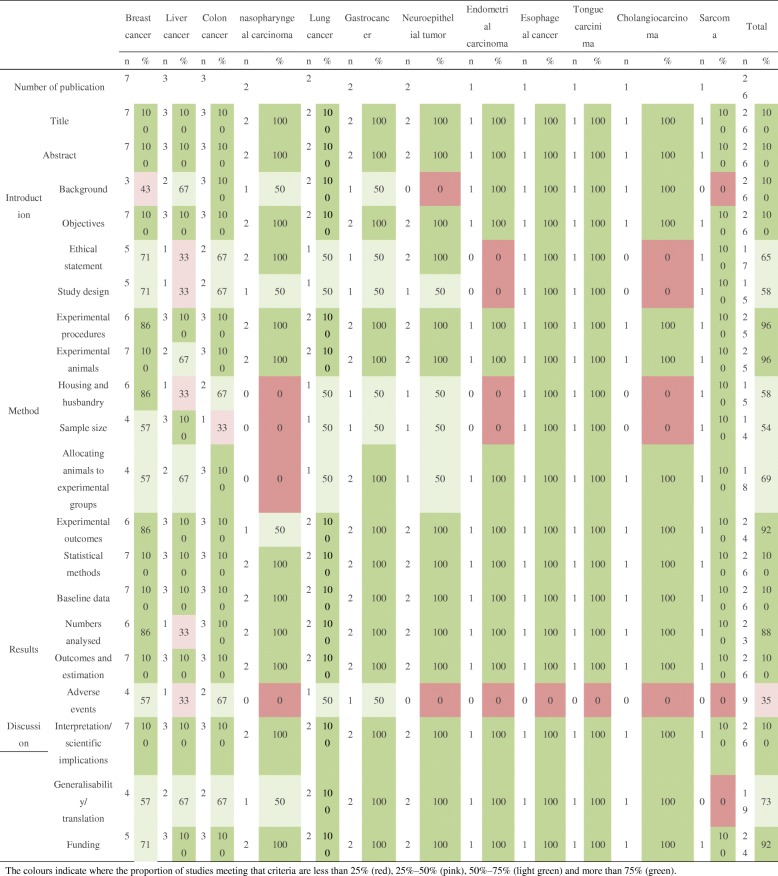
Quality assessment of eligible studies with ARRIVE checklist

The colours indicate where the proportion of studies meeting that criteria are less than 25% (red), 25%–50% (pink), 50%–75% (light green) and more than 75% (green)

### Tumor volume

**Fig. 2:**
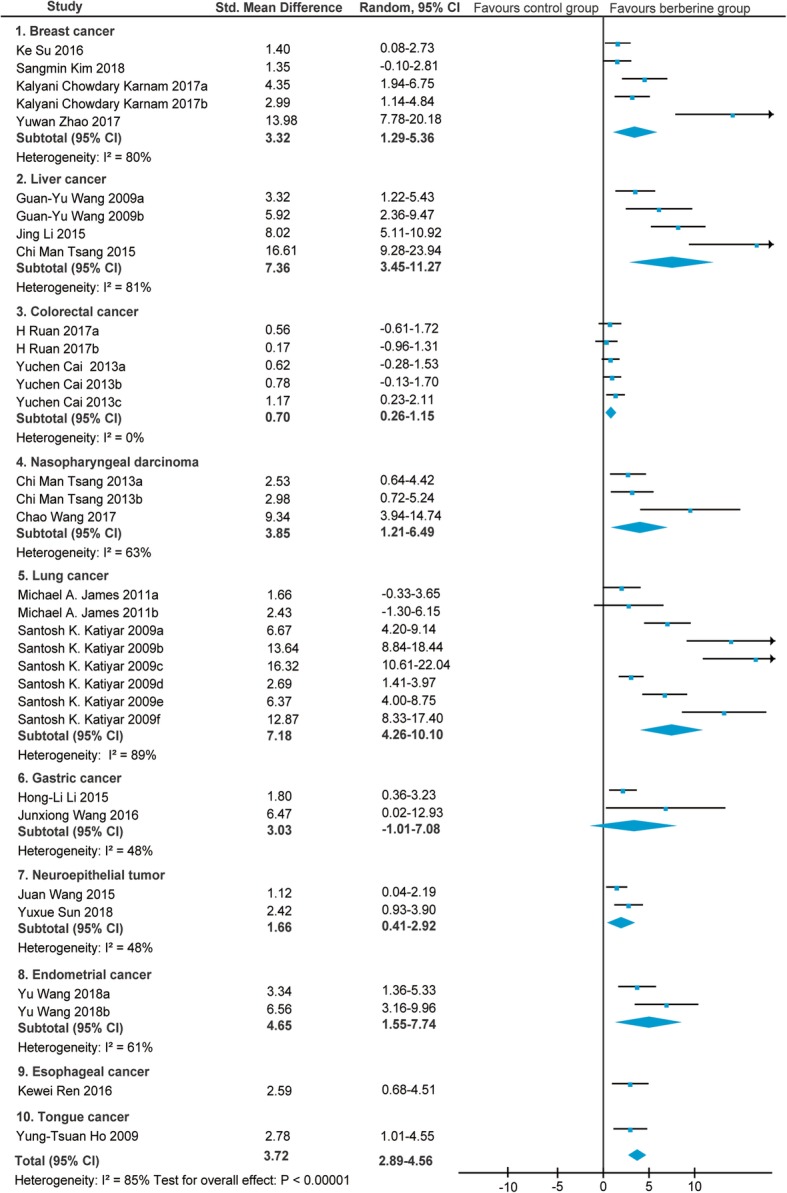
Forest plot of the tumor volume

Of the 26 screened articles [10–35], 20 [11, 13, 14, 16–19, 21–33] reported the relationship between BBR and tumor volume in animals with breast cancer [11, 13, 14, 16], liver cancer [17–19], colorectal cancer [21, 22], nasopharyngeal carcinoma [23, 24], lung cancer [25, 26], gastric cancer [27, 28], neuroepithelial cancer [29, 30], endometrial carcinoma [31], esophageal cancer [32], and tongue cancer [33]. The SMD and the 95%CI in each study are shown in Fig. 2. The pooled SMD remained statistically significant in breast cancer (SMD = 3.32, 95% CI: 1.29, 5.36; Z = 3.2, *p* = 0.001), liver cancer (SMD = 7.36, 95% CI: 3.45, 11.27; Z = 3.69, *p* = 0.0002), colorectal cancer (SMD = 0.70, 95% CI: 0.26, 1.15; Z = 3.10, *p* = 0.002), nasopharyngeal carcinoma (SMD = 3.85, 95% CI: 1.21, 6.49; Z = 2.86, *p* = 0.004), lung cancer (SMD = 7.18, 95% CI: 4.26, 10.10; Z = 4.82, *p* < 0.00001), neuroepithelial tumor (SMD = 1.66, 95% CI:0.41, 2.92; Z = 2.59, *p* = 0.010), and endometrial cancer (SMD = 4.65, 95% CI: 1.55, 7.74; Z = 2.94, *P* = 0.003). The pooled SMD remained statistically insignificant in gastric cancer (SMD = 1.47, 95% CI: − 1.01, 7.08; Z = 1.47, *P* = 0.14). For total studies, the pooled result suggested that the SMD was 3.72 (95% CI: 2.89, 4.56) with statistical significance (Z = 8.73, *p* < 0.00001).

**Fig. 3:**
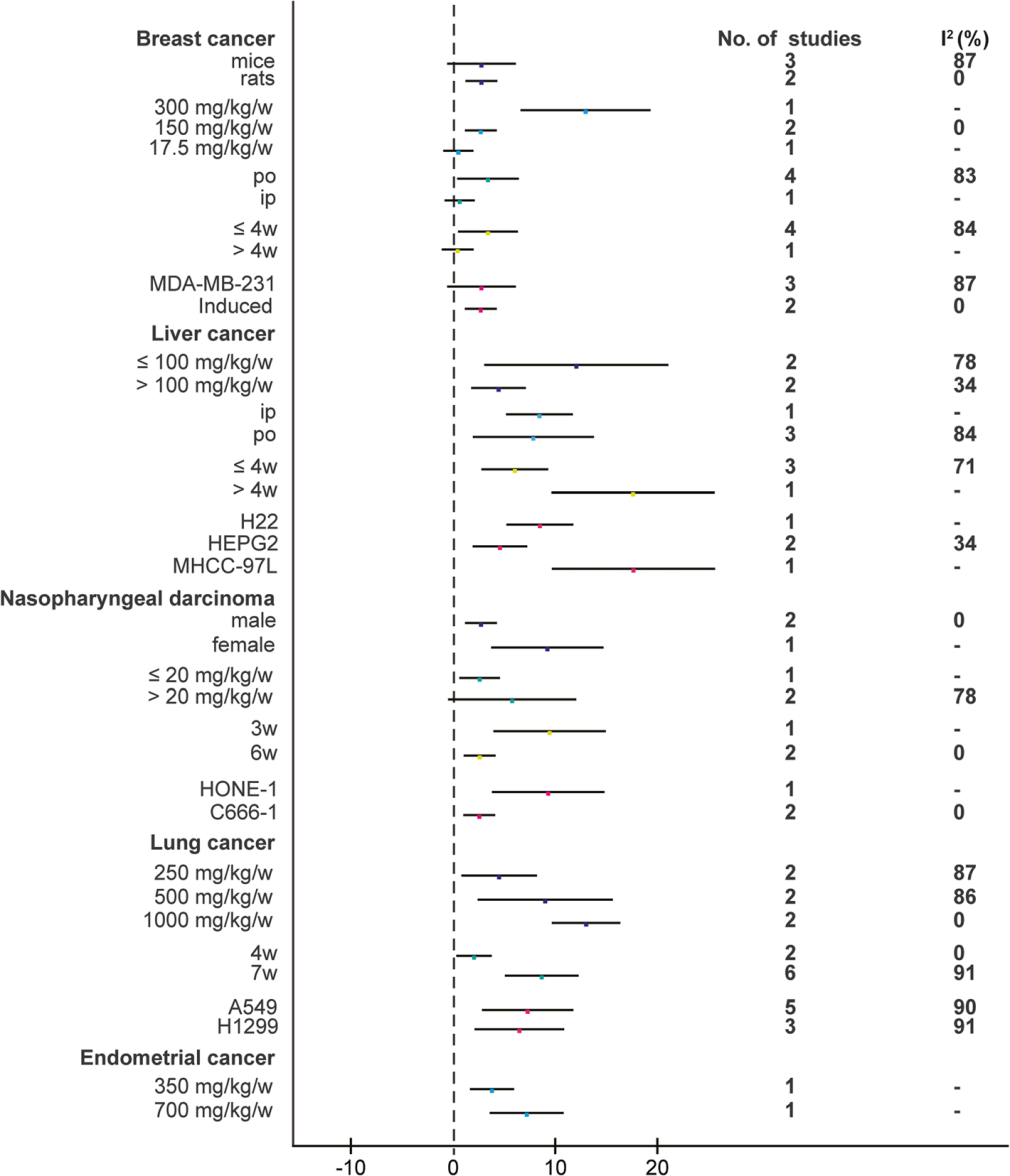
Subgroup analyses of the tumor volume

In view of the obvious heterogeneity (*I*^2^ = 80% for breast cancer; *I*^2^ = 81% for liver cancer; *I*^2^ = 63% for nasopharyngeal carcinoma; *I*^2^ = 89% for lung cancer; *I*^2^ = 61% for endometrial cancer), we conducted a subgroup analysis of different characteristics mainly on the following aspects: gender, animals, BBR dose, administration, duration, and cell lines (Fig. 3). In breast cancer, the BBR dose was a potential influencing factor (*I*^2^ decreased to 0% in one subgroup. Another two *I*^2^ were missing due to the limited study). In liver cancer, the cell line was a potential influencing factor (*I*^2^ decreased to 34% in one subgroup. Another two *I*^2^ were missing due to the limited study). In nasopharyngeal carcinoma, gender, duration, and cell line were potential influencing factors (*I*^2^ decreased to 0% in one subgroup. Another *I*^2^ was missing due to the limited study). In lung cancer, the BBR dose was a potential influencing factor (*I*^2^ decreased to 87, 86, and 0% in three subgroups respectively). In endometrial cancer, no potential influencing factor was filtered.

**Fig. 4:**
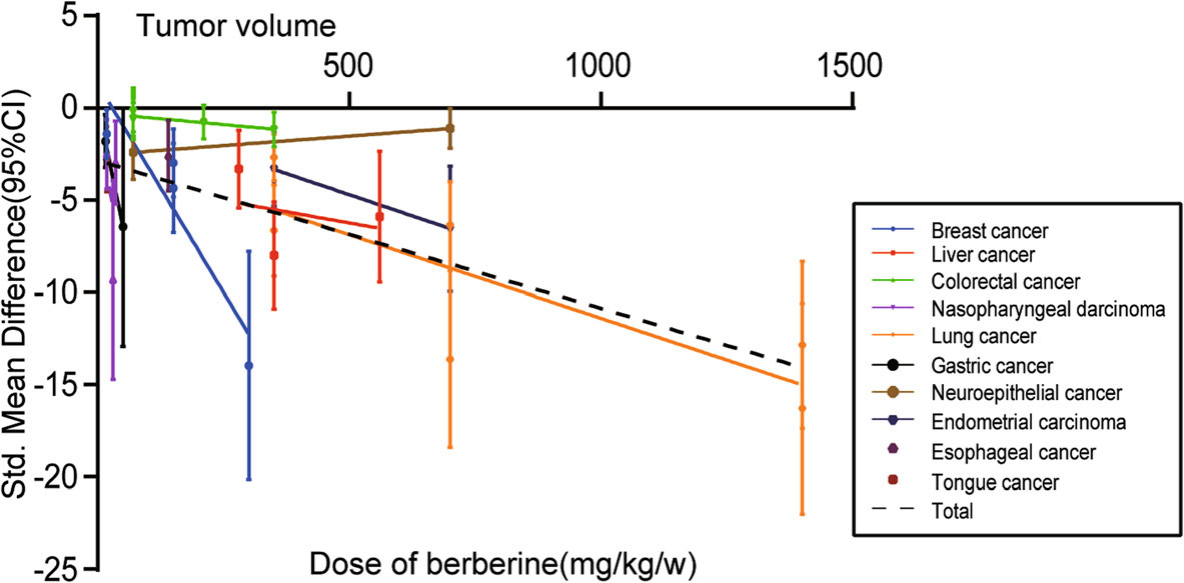
The dose–response relationship of BBR and tumor volume

The dose–response relationship of different cancer types on the relationship between BBR and tumor volume of animals is shown in Fig. 4. For single cancer types, a statistically significant linear relationship in colorectal cancer (Pearson r = − 0.8785, *p* = 0.0499) and lung cancer (Pearson r = − 0.6718, *p* = 0.0459) was observed. For total studies, the SMD values of all studies showed a statistically significant decreasing trend with increasing concentration of BBR (Pearson r = − 0.6717, *p* < 0.0001).

### Tumor weight

**Fig. 5:**
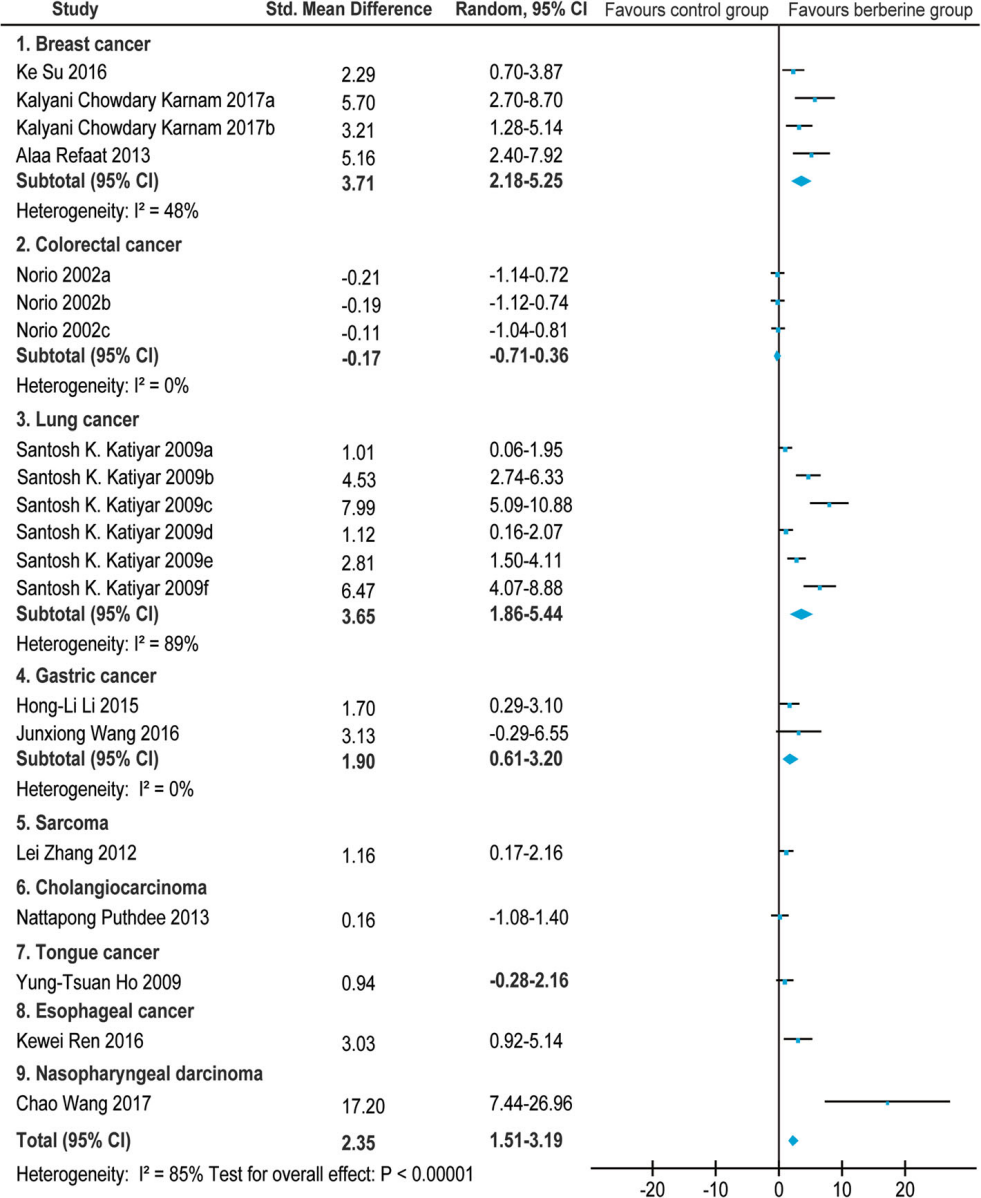
Forest plot of the tumor weight

Of the 26 screened articles [10–35], 12 [12, 14, 16, 20, 23, 26–28, 32–35] reported the relationship between BBR and tumor weight in animals with breast cancer [12, 14, 16], colorectal cancer [20], nasopharyngeal carcinoma [23], lung cancer [26], gastric cancer [27, 28], esophageal cancer [32], and tongue cancer [33]. The SMD and the 95%CI in each study are shown in Fig. 5. The pooled SMD remained statistically significant in breast cancer(SMD = 3.71, 95% CI: 2.18, 5.25; Z = 4.74, *p* < 0.00001), lung cancer(SMD = 3.65, 95% CI: 1.86, 5.44; Z = 4.00, *p* < 0.0001), and gastric cancer(SMD = 1.90, 95% CI: 0.61, 3.20; Z = 2.88, *p* = 0.004). The pooled SMD remained statistically insignificant in colorectal cancer(SMD = − 0.17, 95% CI: − 0.71, 0.36; Z = 0.63, *p* = 0.53). For total studies, the pooled result suggested that the SMD was 2.35(95% CI: 1.51, 3.19) with statistical significance (Z = 5.50, *p* < 0.00001).

**Fig. 6:**
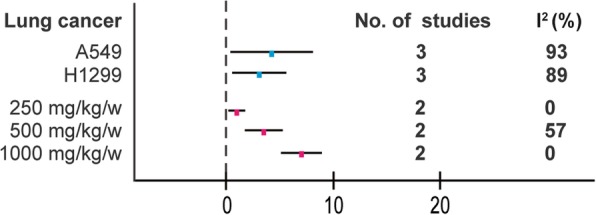
Subgroup analyses of the tumor weight

In view of the obvious heterogeneity(*I*^*2*^ = 89% for lung cancer), we conducted a subgroup analysis of different characteristics mainly on the following aspects: dose of BBR and cell lines(Fig. 6). In lung cancer, the dose of BBR was a potential influencing factor (*I*^*2*^ decreased to 0, 57, and 0% in three subgroups respectively).

**Fig. 7:**
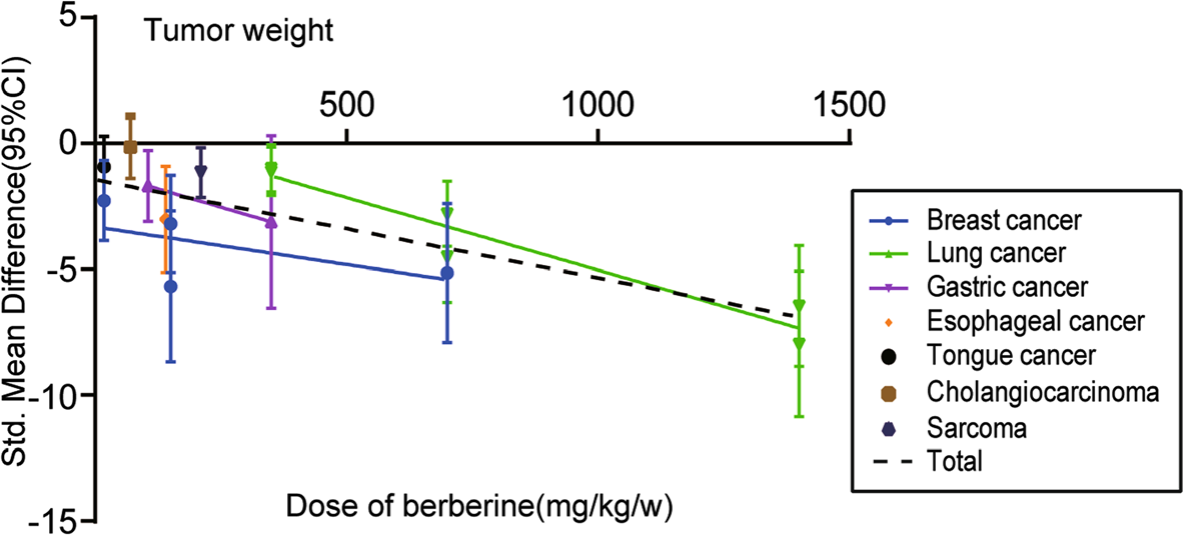
The dose–response relationship of BBR and tumor weight

The dose–response relationship of different cancer types on the relationship between BBR and tumor weight of animals is shown in Fig. 7. For single cancer types, a statistically significant linear relationship in lung cancer (Pearson r = − 0.9623, *p* = 0.0021) was observed. For total studies, the SMD values of all studies showed a statistically significant decreasing trend with increasing concentration of BBR (Pearson r = − 0.7704, *p* < 0.0005).

### Tumor vessel density

**Fig. 8:**
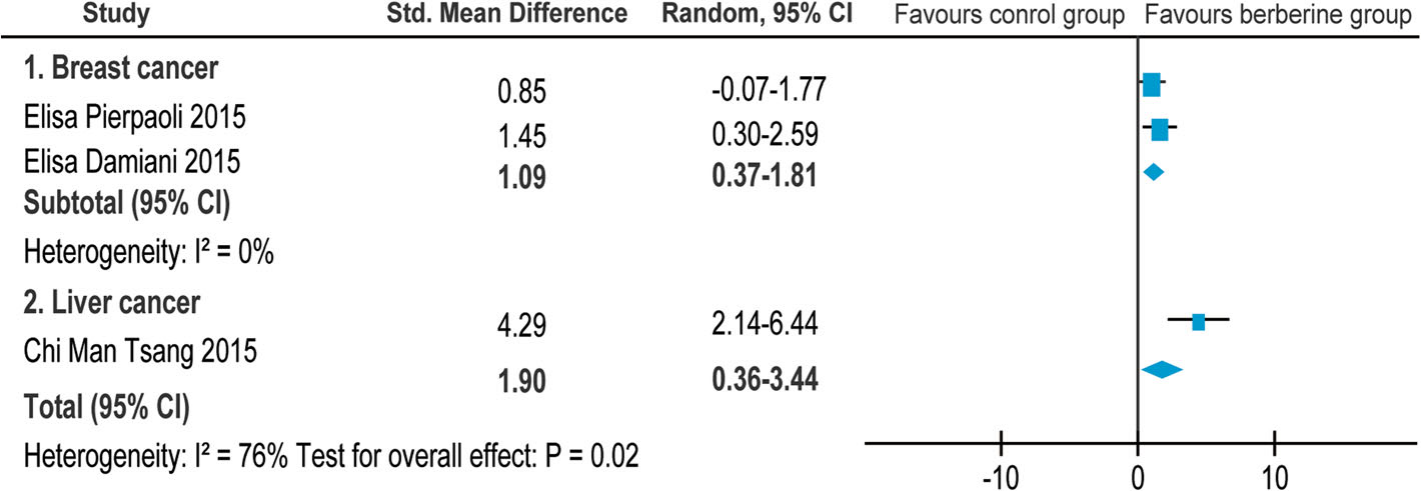
Forest plot of the tumor vessel density

Of the 26 screened articles [10–35], 3 [10, 15, 19] reported the relationship between BBR and tumor vessel density in animals with breast cancer [10, 15] and liver cancer [19]. The SMD and the 95%CI in each study are shown in Fig. 8. The pooled SMD remained statistically significant in breast cancer(SMD = 1.09, 95% CI: 0.37, 1.81; Z = 2.96, *p* = 0.003). For total studies, the pooled result suggested that the SMD was 4.29(95% CI: 2.14, 6.44) with statistical significance(Z = 3.92, *p* < 0.00001).

No statistical heterogeneity was observed (*I*^*2*^ = 0% for breast cancer).

**Fig. 9:**
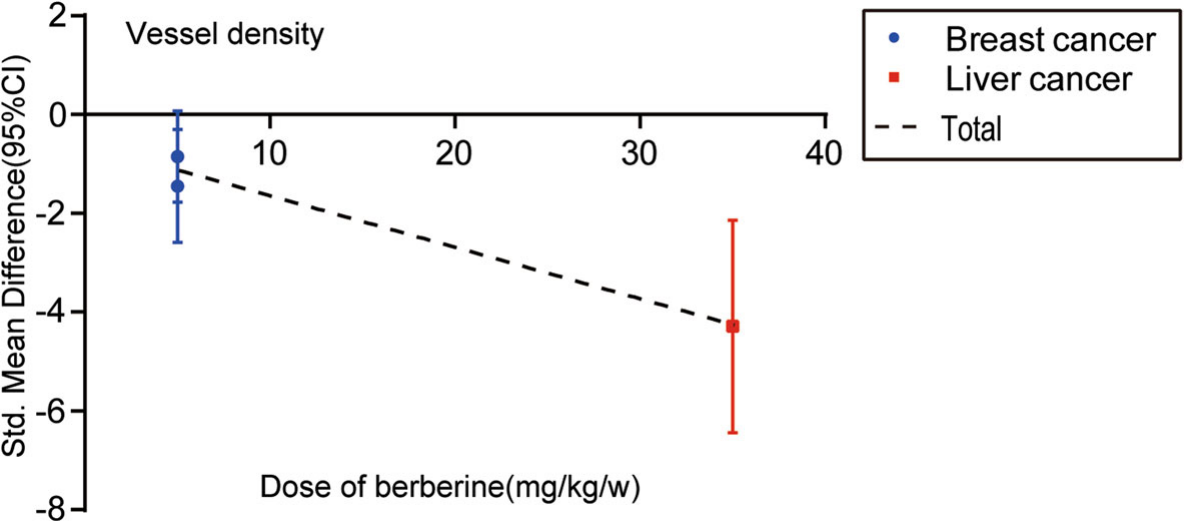
The dose–response relationship of BBR and tumor vessel density

The dose–response relationship of different cancer types on the relationship between BBR and tumor weight of animals is shown in Fig. 9. For single cancer types, no linear relationship was concluded because of the limited studies. For total studies, the SMD values of all studies showed no statistically significant trend(Pearson r = − 0.9866, *p* = 0.1044).

### Body weight

**Fig. 10:**
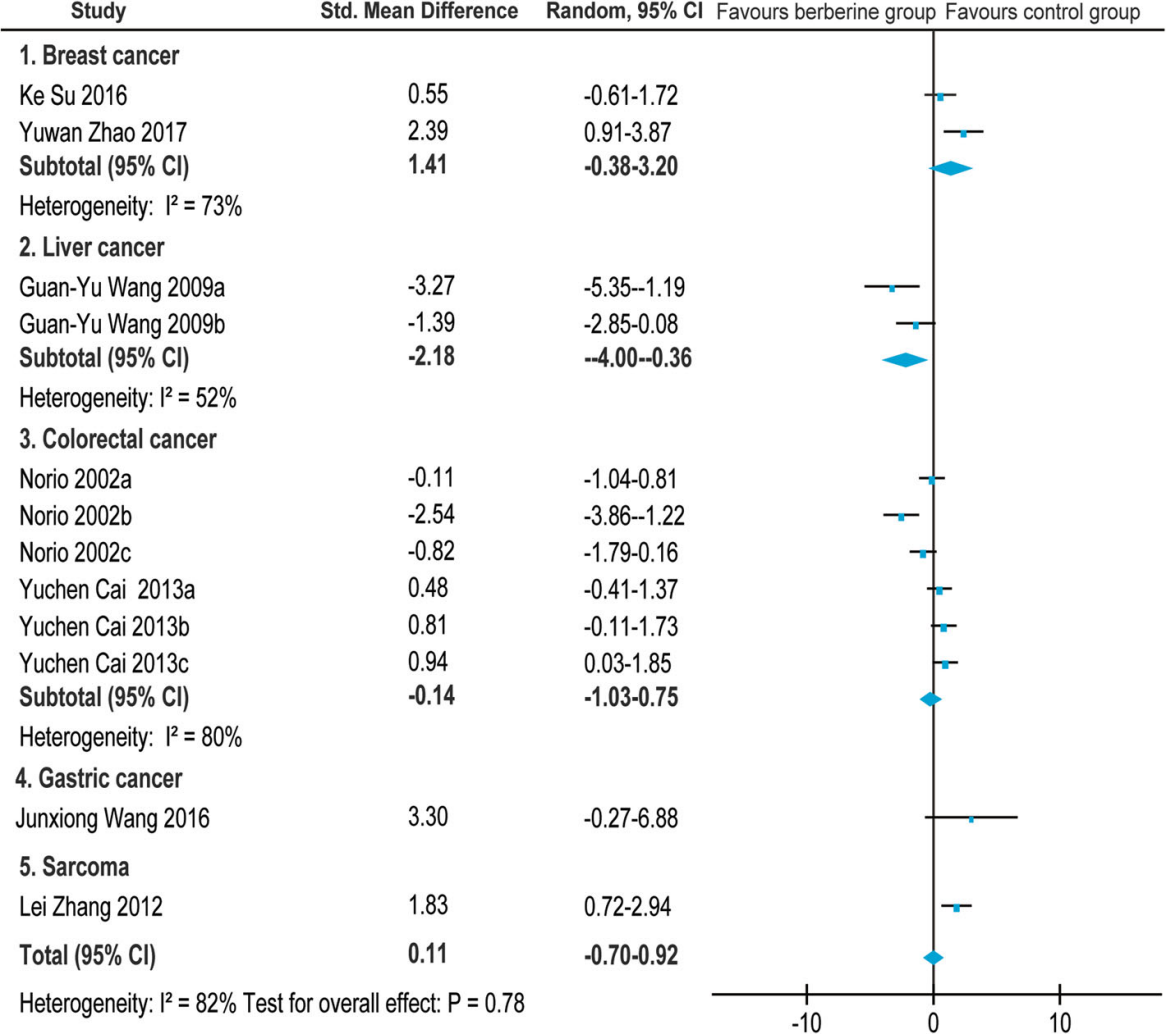
Forest plot of the body weight

Of the 26 screened articles [10–35], 7 [11, 16, 17, 20, 22, 27, 35] reported the relationship between BBR and body weight in animals with breast cancer [11, 16], liver cancer [17], colorectal cancer [20, 22], gastric cancer [27], and sarcoma [35]. The SMD and the 95%CI in each study are shown in Fig. 10. The pooled SMD remained statistically significant in liver cancer(SMD = − 2.18, 95% CI: − 4.00, − 0.36; Z = 2.35, *p* = 0.02). The pooled SMD remained statistically insignificant in breast cancer(SMD = 1.41, 95% CI: − 0.38, 3.20; Z = 1.54, *p* = 0.12) and colorectal cancer(SMD = − 0.14, 95% CI: − 1.03, 0.75; Z = 0.30, *p* = 0.76). For total studies, the pooled result suggested that the SMD was 0.11(95% CI: − 0.70, 0.92) with statistical significance(Z = 0.27, *p* = 0.78).

**Fig. 11:**
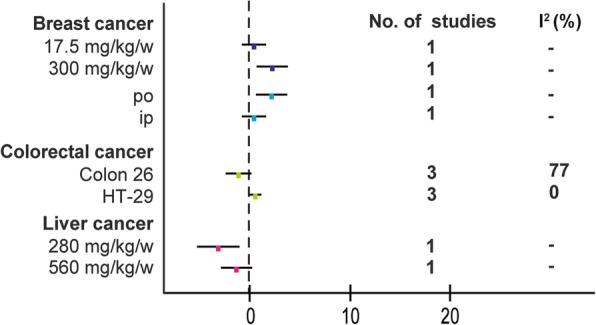
Subgroup analyses of the body weight

In view of the obvious heterogeneity(*I*^*2*^ = 73% for breast cancer; *I*^*2*^ = 80% for colorectal cancer; *I*^*2*^ = 52% for liver cancer), we conducted a subgroup analysis of different characteristics mainly on the following aspects: dose of BBR, administration, and cell lines(Fig. 11). *I*^*2*^ were missing in breast cancer group and liver cancer group due to limited studies. No potential influencing factor was found in colorectal cancer group.

**Fig. 12:**
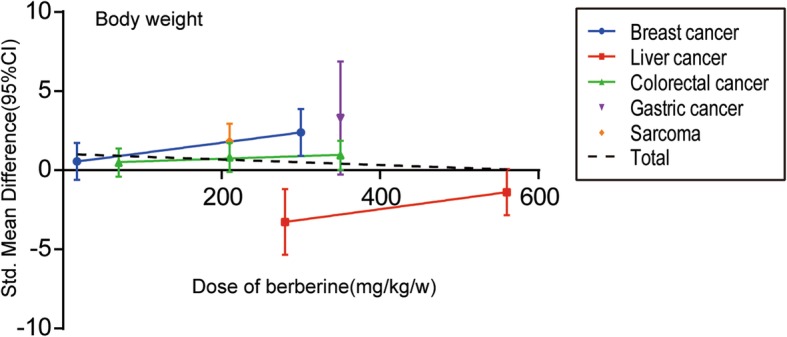
The dose–response relationship of BBR and body weight

The dose–response relationship of different cancer types on the relationship between BBR and body weight of animals is shown in Fig. 12. For single cancer types, no statistically significant linear relationship was found. For total studies, the SMD values of all studies showed no statistically significant trend(Pearson r = − 0.1440, *p* = 0.7116).

### Publication bias and sensitivity analysis

**Fig. 13:**
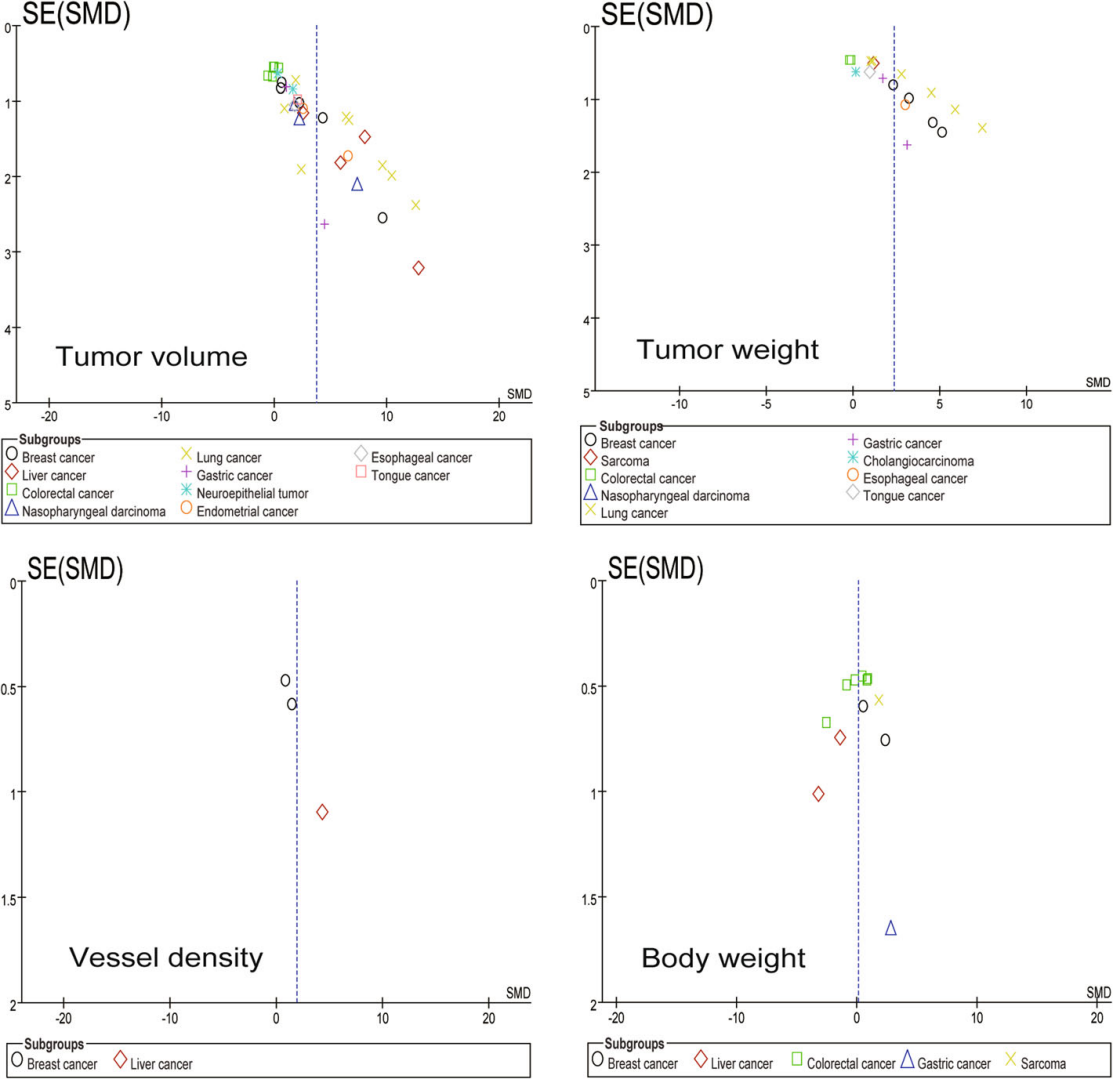
Funnel plot of tumor volume studies, tumor weight studies, tumor vessel density studies, and body weight studies

The publication bias evaluation for the meta-analysis of tumor volume, tumor weight, tumor vessel density, and body weight is shown in Fig. 13. These funnel plots showed that most of the studies are in the upper part of the inverted funnel and approximately symmetrical, suggesting that the publication bias was unobvious.

**Fig. 14:**
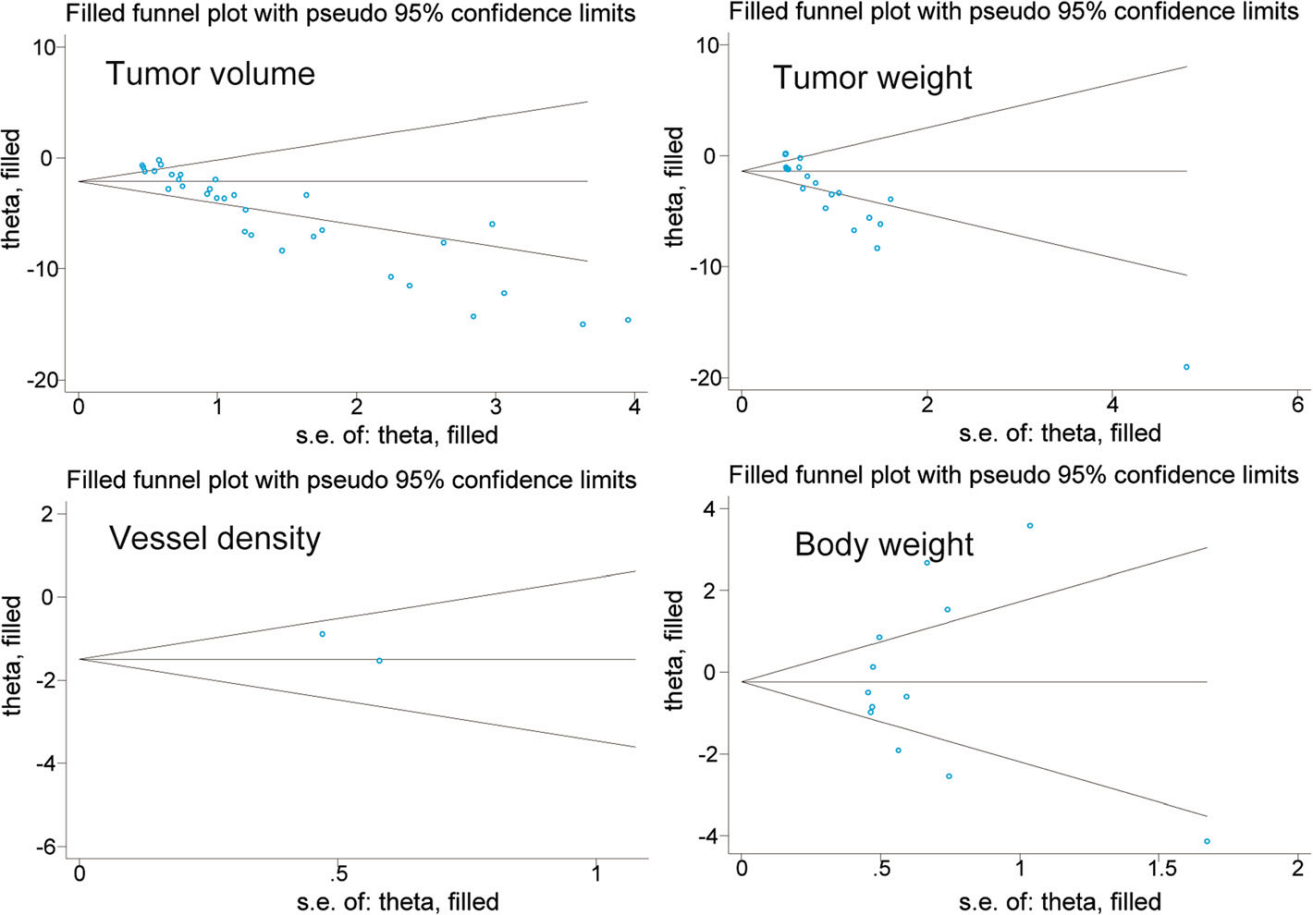
Metatrim plot of tumor volume studies, tumor weight studies, tumor vessel density studies, and body weight studies

**Fig. 15:**
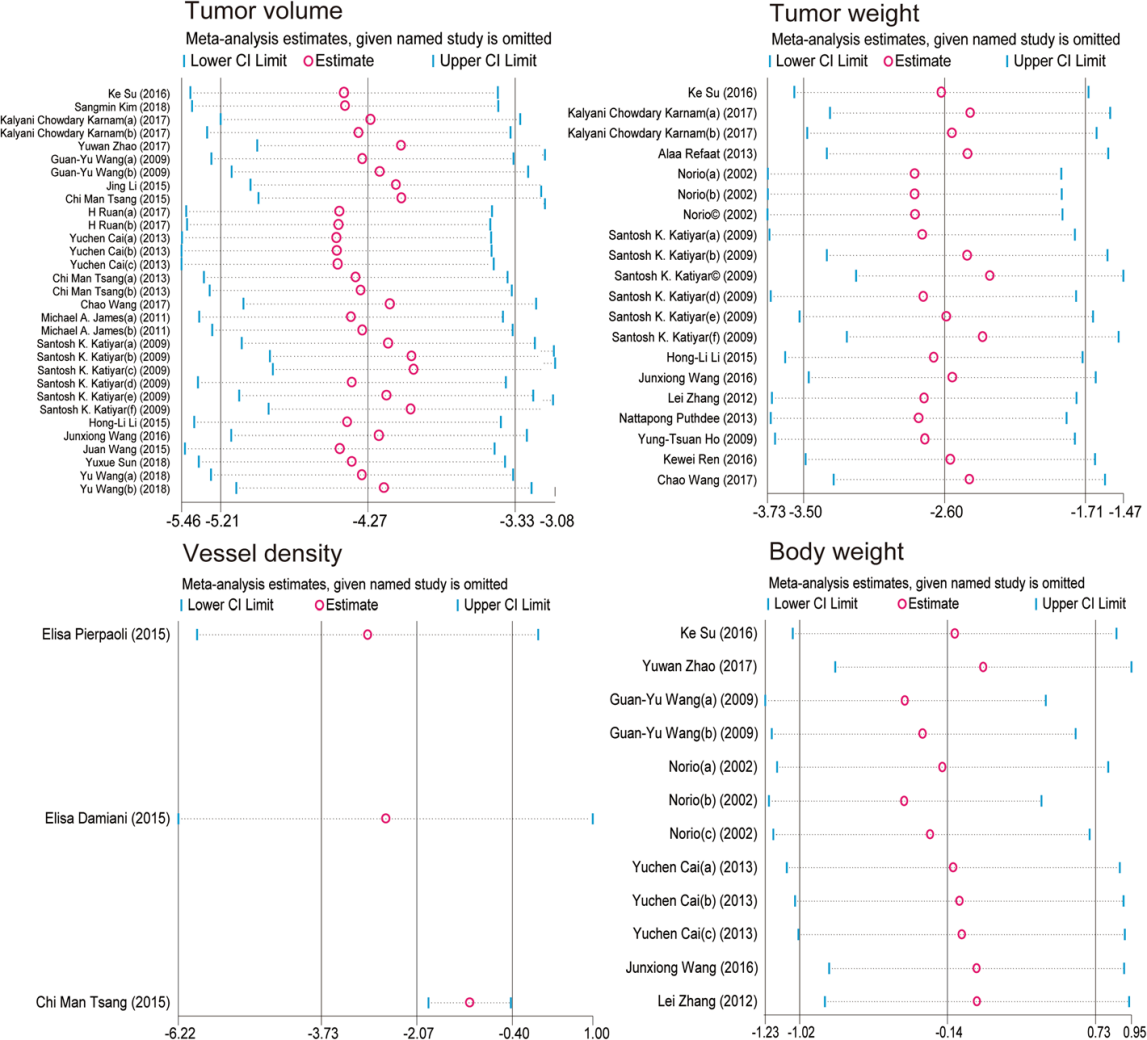
Metaninf plot of tumor volume studies, tumor weight studies, tumor vessel density studies, and body weight studies

A sensitivity analysis was performed to assess the stability of our results in terms of tumor volume, tumor weight, tumor vessel density, and body weight. The trim method was used, and the results did not show considerable changes between the previous and new SMDs (Fig. 14). Next, we deleted one individual study at a time, and the results of the rest of the studies were checked for any reversal. The statistical outcomes showed that the pooled SMDs were all still significant although one study was excluded (Fig. 15).

### Molecular pathways and proteins

Among these included studies, a wide range of molecular targets, which are essential for the anti-cancer effect of BBR, was revealed. Except for three articles [15, 33, 35] that did not involve the discussion of molecular mechanisms, the remaining 23 articles [10–14, 16–32, 34] analyzed the anti-tumor mechanism of BBR. The pharmacological effects of BBR was summarized into five aspects: proliferation(including apoptosis, autophagy, cell cycle arrest, and others), intracellular oxidative stress, inflammation, angiogenesis, and migration. Table 3 shows how BBR works in different scenarios of multiple types of cancers. In addition, in order to understand the anticancer mechanism more clearly and deeply, Table 4 shows the clustering analysis of the common molecular pathways and target proteins between studies.

**Table 3:** Molecular pathways and proteins in different cancers

Molecular Pathway	Proteins	Functional clustering
Breast cancer		
↑ caspase-9/cytochrome c-mediated apoptosis [11]; TRAIL(TNF-related apoptosis-inducing ligand)-mediated apoptosis [12] ↓ cell proliferation [14]	↑ caspase-3 [11, 12]; caspase-9, ClvC-3, Bax, Ligase4 [11]; PARP, P53 [12] ↓ Bcl-2 [11]; P65, Mcl-1 [12]; PCNA [14]	Proliferation(including apoptosis)
↑ intracellular reactive oxygen species (ROS) levels [14]	↑ MDA [14] ↓ SOD, CAT, GSH, Vit-C [14]	Intracellular oxidative stress
↓ inflammation [14]	↓ IL-1β, IL-6, TNF-α, NF-kB [14]	Inflammation
↓ TGF-β1-induced cell migration [13]; vasodilator-stimulated phosphoprotein (VASP)-induced cell migration [16]	↓TGF-β1, MMP-2, MMP-9 [13] No effect: VASP [16]	Migration
Liver cancer		
↑ Fas-mediated apoptosis [17] ↓ arachidonic acid metabolic pathway [18]; Id-1-induced cell proliferation [19]	↑Fas, P53, caspase-3, caspase-8, caspase-9 [17] ↓ PGE2, cPLA2, COX-2 [18]; Id-1 [19] No effect: caspase-3, caspase-9 [18]	Proliferation(including apoptosis)
↓ Id-1-induced angiogenesis [19]	↓ Id-1, VEGF, HIF-1α [19]	Angiogenesis
↓ Id-1-induced migration [19]	↓Id-1 [19]	Migration
Colon cancer		
↓ β-catenin - induced proliferation by binding RXR [21]; cell proliferation by inducing the G2/M phase arrest and down-regulated the expression of the related cyclins [22]	↑ c-Cbl, p21^WAF1/CIP1^ [21] ↓ Cdc2 [21, 22]; PCNA, β-catenin, Ki-67, c-myc, RXRα [21]; cyclin B1, cdc25c [22]	Proliferation(including cell cycle arrest)
Nasopharyngeal carcinoma		
↓cell proliferation via an Epstein-Barr virus nuclear antigen 1(EBNA1)-dependent mechanism [23]; cell proliferation by inhibiting STAT3 activation [24]	↑ Cleaved PARP [24] ↓ Mcl-1, p-STAT3 [23, 24]; EBNA1 [23]	Proliferation
Lung cancer		
↑ G1 cell cycle arrest [25]; P53-Induced growth inhibition and apoptosis [26] ↓cell proliferation via MAPK pathways [25]	↑ P53 [25, 26]; Bax, Bak, caspase-3 [26] ↓ p-Akt, p-CREB, p-MAPK, cyclin B1 [25]; Bcl-2, Bcl-xl [26]	Proliferation(including apoptosis and cell cycle arrest)
Gastric cancer		
↑ apoptosis and cell cycle arrest via inhibiting EGFR signaling [27] ↓ cell proliferation via MAPK pathways [28]	↓pERK [27, 28]; pAKT, pSTAT3, pNFκB, NFκB, Bcl-xL, cyclin D1 [27]; p-P38 MAPK, p-JNK, IL-8 [28]	Proliferation(including apoptosis and cell cycle arrest)
Neuroepithelial cancer		
↑ ERK1/2-mediated impairment of mitochondrial aerobic respiration and autophagy [30] ↓cancer growth by suppressing Hedgehog signaling pathway [29]	↑ C-parp-1, LC3II [30] ↓ Gli1, PTCH1 [29]; Ki-67, p-ERK1/2 [30]	Proliferation(including autophagy)
Endometrial carcinoma		
↓ cell growth via miR-101/COX-2 [31]	↓ COX-2, PGE2 [31]	Proliferation
↓ cell metastasis via miR-101/COX-2 [31]	↓ COX-2, PGE2 [31]	Migration
Esophageal cancer		
↑ cell growth inhibition, apoptosis and cell cycle arrest at G2/M phase [32]	↑ P21, P27, P53, cleaved-PARP, caspase-3, Bax [32] ↓ PI3K, Rac, p-JAK2, p-STAT3, Wnt3a, β-catenin, Bcl-2, Mcl-1, XIAP, Ki-67, cyclin B, cyclin D, cyclin E, CDK1, CDK2, CDK4, CDK6 [32]	Proliferation(including apoptosis and cell cycle arrest)
Cholangiocarcinoma		
↑ G1 cell cycle arrest [34] ↓ cell proliferation [34]	↓ PCNA, cyclin D1, cyclin E [34]	Proliferation(including cell cycle arrest)

**Table 4:** Cluster analysis of molecular pathways and proteins in different cancers

Functional clustering	Molecular Pathway	Proteins
Proliferation(apoptosis)	Breast cancer: ↑ caspase-9/cytochrome c-mediated apoptosis [11]; TRAIL(TNF-related apoptosis-inducing ligand)-mediated apoptosis [12] Liver cancer: ↑ Fas-mediated apoptosis [17]; ↓ arachidonic acid metabolic pathway [18] Lung cancer: ↑ P53-Induced growth inhibition and apoptosis [26] Gastric cancer: ↑ apoptosis via inhibiting EGFR signaling [27] Esophageal cancer: ↑ cell growth inhibition and apoptosis [32]	**↑** caspase-3 [11, 12, 17, 26, 32]; P53 [12, 17, 25, 26, 32]; Bax [11, 26, 32]; caspase-9 [11, 17]; PARP [12, 32]; ClvC-3, Ligase4 [11]; Fas [17]; caspase-8 [17]; Bak [26]; P21, P27 [32] **↓** Bcl-2 [11, 26, 32]; Mcl-1 [12, 32]; Bcl-xl [26, 28]; pERK [27, 28]; pSTAT3 [28, 32]; P65 [12]; PGE2, cPLA2, COX-2 [18]; pAKT, pNFκB, NFκB [28]; PI3K, Rac, p-JAK2, Wnt3a, β-catenin, XIAP, Ki-67 [32] No effect: caspase-3, caspase-9 [18]
Proliferation(autophagy)	Neuroepithelial cancer: ↑ ERK1/2-mediated impairment of mitochondrial aerobic respiration and autophagy [30]	↑ C-parp-1, LC3II [30] ↓ Ki-67, p-ERK1/2 [30]
Proliferation(cell cycle arrest)	Colon cancer: ↓ cell proliferation by inducing the G2/M phase arrest and down-regulated the expression of the related cyclins [22] Lung cancer: ↑ G1 cell cycle arrest [25] Gastric cancer: ↑ cell cycle arrest via inhibiting EGFR signaling [27] Esophageal cancer: ↑ cell cycle arrest at G2/M phase [32] Cholangiocarcinoma: ↑ G1 cell cycle arrest [34]	↓ cyclin B1 [22, 25, 32]; cyclin D1 [27, 32, 34]; cyclin E [32, 34]; Cdc2 [22]; cdc25c [22]; CDK1, CDK2, CDK4, CDK6 [32]
Proliferation(others)	Breast cancer: ↓ cell proliferation [14] Liver cancer: ↓ Id-1-induced cell proliferation [19] Colon cancer: ↓ β-catenin - induced proliferation by binding RXR [21] Nasopharyngeal carcinoma: ↓ cell proliferation via an Epstein-Barr virus nuclear antigen 1(EBNA1)-dependent mechanism [23]; ↓ cell proliferation by inhibiting STAT3 activation [24] Lung cancer: ↓cell proliferation via MAPK pathways [25] Gastric cancer: ↓ cell proliferation via MAPK pathways [28] Neuroepithelial cancer: ↓cancer growth by suppressing Hedgehog signaling pathway [29] Endometrial carcinoma: ↓ cell growth via miR-101/COX-2 [31] Cholangiocarcinoma: ↓ cell proliferation [34]	↑ c-Cbl, p21^WAF1/CIP1^ [21]; Cleaved PARP [24] ↓ PCNA [14, 21, 34]; Mcl-1, p-STAT3 [23, 24]; p-MAPK [25, 28]; Id-1 [19]; β-catenin, Ki-67, c-myc, RXRα [21]; EBNA1 [23]; p-Akt, p-CREB [25]; p-JNK, IL-8 [28]; Gli1, PTCH1 [29]; COX-2, PGE2 [31]
Intracellular oxidative stress	Breast cancer: ↑ intracellular reactive oxygen species (ROS) levels [14]	↑ MDA [14] ↓ SOD, CAT, GSH, Vit-C [14]
Inflammation	Breast cancer: ↓ inflammation [14]	↓ IL-1β, IL-6, TNF-α, NF-kB [14]
Angiogenesis	Liver cancer: ↓ Id-1-induced angiogenesis [19]	↓ Id-1, VEGF, HIF-1α [19]
Migration	Breast cancer: ↓ TGF-β1-induced cell migration [13]; vasodilator-stimulated phosphoprotein (VASP)-induced cell migration [16] Liver cancer: ↓ Id-1-induced migration [19] Endometrial carcinoma: ↓ cell metastasis via miR-101/COX-2 [31]	↓TGF-β1, MMP-2, MMP-9 [13]; Id-1 [19]; COX-2, PGE2 [31] No effect: VASP [16]

The most frequently studied pathways were on cell proliferation and 19 articles focused on this mechanism. Seven of these studies involved tumor cell apoptosis pathways (breast cancer [9, 12], liver cancer [17, 18], lung cancer [26], gastric cancer [27], esophageal cancer [32]), one involved autophagy pathways (neuroepithelial cancer [30]), and five involved cell cycle arrest pathways (colon cancer [22], lung cance [25], gastric cancer [27], esophageal cancer [32], cholangiocarcinoma [34]). The second most common frequently studied pathways were on cell migration. Four articles in three cancers studied the relationship between BBR and tumor cell migration (breast cancer [13, 16], liver cancer [19], endometrial carcinoma [31]). There was only one study reported the relationship between BBR and intracellular oxidative stress (breast cancer [14]), inflammation (breast cancer [14]), and angiogenesis (liver cancer [19]) respectively.

## Discussion

We performed a systematic review and meta-analysis to systematically evaluate the efficacy and adverse effect of BBR on various cancers. The results showed that BBR could inhibit the growth of a variety of cancers in vivo, especially in breast cancer (SMD = 3.32, 95% CI: 1.29, 5.36 in tumor volume; SMD = 3.71, 95% CI: 2.18, 5.25 in tumor weight; SMD = 1.09, 95% CI: 0.37, 1.81 in tumor vessel density) and lung cancer (SMD = 7.36, 95% CI: 3.45, 11.27 in tumor volume; SMD = 3.65, 95% CI: 1.86, 5.44 in tumor weight). Evidence for the benefit of BBR was not sufficient for gastric cancer (SMD = 1.47, 95% CI: − 1.01, 7.08 in tumor volume) and colorectal cancer (SMD = − 0.17, 95% CI: − 0.71, 0.36 in tumor weight). BBR showed a dose–response relationship in tumor volume and weight (Pearson r = − 0.6717 and − 0.7704, with *p* < 0.0001 and *p* < 0.0005, respectively). At the same time, dose was an important influencing factor for heterogeneity from the subgroup analysis. The change in body weight of experimental animals was used as an indicator of the adverse effects of BBR. The above results indicated that no statistically significant difference was observed in terms of body weight under the effect of BBR (SMD =0.11, 95% CI: − 0.70, 0.92).

In the past 3 years, numerous studies have attempted to elucidate the relationship between BBR and breast/lung cancer. By using molecular modeling and in vitro studies, BBR significantly reduced EGFR and AKT phosphorylation and may be a useful alternative to lapatinib, an EGFR inhibitor which can cause acquired drug resistance in breast cancer patients [36]. BBR lowers blood sugar, increases insulin sensitivity, and corrects lipid metabolism disorders; it may reduce the incidence of breast cancer [37]. Single-drug BBR has an obvious inhibitory effect on lung cancer cells; BBR can inhibit doxorubicin (DOX)-mediated STAT3 activation and sensitize lung cancer cells to the cytotoxic effects of DOX treatment. Given the widespread clinical application of BBR and its low toxicity, our findings are important for the development of a new combination of BBR and DOX for the treatment of lung cancer [38]. In addition to medical treatment, BBR has protective effects on radiation-induced lung injury via intercellular adhesion molecular-1 and transforming growth factor-beta-1 in patients with lung cancer [39].

Although, in the present study, the therapeutic effect of BBR in colorectal and gastric cancer required more evidence, numerous studies have confirmed the gain effect of BBR combined with chemotherapy in recent years. Latest research shows that the combination of the second generation Hsp90 inhibitor NVP-AUY922 and BBR therapy could inhibit a variety of oncogenic signaling pathways of colorectal cancer [40]. Another study showed that BBR as an adjunctive therapeutic agent could attenuate chemical resistance during gastric cancer treatment. The combination of 5-FU and BBR showed synergistic inhibition of survivin and STAT3 levels, thereby enhancing the death of gastric cancer cells [41]. In addition to the 5-FU-based chemotherapy regimen, BBR treatment reduced cisplatin resistance in gastric cancer cells by modulating the miR-203/Bcl-w apoptotic axis [42].

In the present study, body weight index was used to evaluate the growth of experimental animals to indirectly evaluate the adverse effects of BBR. However, studies have shown that BBR could induce weight loss in rodents [43, 44] and humans [45, 46]. In recent years, research has reported that BBR affected body weight by upregulating AMPK and UCP3 expression to control energy expenditure [47]. Therefore, the toxic side effects of BBR cannot be objectively and accurately evaluated by the change of body weight alone.

## Limitations

There were some limitations to our analysis that deserve discussion. First, we observed considerable heterogeneity between the studies when analyzing tumor volume, tumor weight, and body weight. Although subgroup analysis (Figs. 3, 5, and 11) was performed, some *I*^*2*^ were missing because of the limited studies. Secondly, generally speaking, obviously significant publication bias was not found based on the funnel plot (Fig. 13). However, poor symmetry of the funnel plot on tumor volume suggested more high-quality researches should be included. Thirdly, although PubMed, Embase, Springer, and Cochrane databases had been carefully and comprehensively searched, articles selected for each cancer type were still small which could lead to bias. Fourthly, the anticancer effects of berberine in humans were not identified clearly and further studies in humans were needed to develop it as an anticancer agent.

## Conclusion

BBR exerted anti-tumor effects in a variety of tumors in vivo, especially for breast cancer and lung cancer. However, evidence was still insufficient in colorectal cancer and gastric cancer. One of its anti-tumor mechanisms was anti-angiogenesis. There was a dose-response relationship in the anti-tumor effects.

## Data Availability

All data generated or analysed during this study are included in this published article.

## Abbreviations

BBR: Berberine
BW: Body Weight
CI: Confidence Interval
DMSO: Dimethyl Sulfoxide
EBNA1: Epstein-Barr virus nuclear antigen 1
ROS: Reactive Oxygen Species
SD: Standard Difference
SMD: Standard Mean Difference
TRAIL: TNF-related apoptosis-inducing ligand
TV: Tumor Volume
TW: Tumor Weight
VASP: Vasodilator-Stimulated Phosphoprotein
VD: Vessel Density
